# Preeclampsia: Novel Mechanisms and Potential Therapeutic Approaches

**DOI:** 10.3389/fphys.2018.00973

**Published:** 2018-07-25

**Authors:** Zaher Armaly, Jimmy E. Jadaon, Adel Jabbour, Zaid A. Abassi

**Affiliations:** ^1^Department of Nephrology, EMMS Nazareth Hospital, Galilee Faculty of Medicine, Bar-Ilan University, Ramat Gan, Israel; ^2^Department of Obstetrics and Gynecology, EMMS Nazareth Hospital, Galilee Faculty of Medicine, Bar-Ilan University, Ramat Gan, Israel; ^3^Laboratory Medicine, EMMS Nazareth Hospital, Galilee Faculty of Medicine, Bar-Ilan University, Ramat Gan, Israel; ^4^Department of Physiology, The Ruth and Burce Rappaport Faculty of Medicine, Technion–IIT, Haifa, Israel; ^5^Department of Laboratory Medicine, Rambam Health Campus, Haifa, Israel

**Keywords:** preeclampsia, maternity, fetus, endothelium, kidney, placental growth factor (PlGF), soluble growth factor receptor-sFlt, endoglin

## Abstract

Preeclampsia is a serious complication of pregnancy where it affects 5–8% of all pregnancies. It increases the morbidity and mortality of both the fetus and pregnant woman, especially in developing countries. It deleteriously affects several vital organs, including the kidneys, liver, brain, and lung. Although, the pathogenesis of preeclampsia has not yet been fully understood, growing evidence suggests that aberrations in the angiogenic factors levels and coagulopathy are responsible for the clinical manifestations of the disease. The common nominator of tissue damage of all these target organs is endothelial injury, which impedes their normal function. At the renal level, glomerular endothelial injury leads to the development of maternal proteinuria. Actually, peripheral vasoconstriction secondary to maternal systemic inflammation and endothelial cell activation is sufficient for the development of preeclampsia-induced hypertension. Similarly, preeclampsia can cause hepatic and neurologic dysfunction due to vascular damage and/or hypertension. Obviously, preeclampsia adversely affects various organs, however it is not yet clear whether pre-eclampsia *per se* adversely affects various organs or whether it exposes underlying genetic predispositions to cardiovascular disease that manifest in later life. The current review summarizes recent development in the pathogenesis of preeclampsia with special focus on novel diagnostic biomarkers and their relevance to potential therapeutic options for this disease state. Specifically, the review highlights the renal manifestations of the disease with emphasis on the involvement of angiogenic factors in vascular injury and on how restoration of the angiogenic balance affects renal and cardiovascular outcome of Preeclamptic women.

## Introduction

Preeclampsia (PE) is a profound complication of pregnancy, where it affects 3–8% of all pregnancies and dramatically increases the risk of all-cause mortality, especially in women who experienced early, severe, preterm episode (Backes et al., 2011; Jim and Karumanchi, 2017). Preeclampsia negatively affects both the mother and fetus (**Table 1**). Concerning the latter, preeclampsia may cause intra-uterine fetal growth restriction (IUGR), placental abruption, preterm delivery and associated complications including, neonatal respiratory distress syndrome, cerebral palsy, necrotizing enterocolitis retinopathy of prematurity and even perinatal death (**Table 1**; Backes et al., 2011). Besides its deleterious impact on the fetus, preeclampsia also affects the pregnant woman, where it is associated with hypertension, kidney damage, liver injury/failure, central nervous system (CNS) damage, stroke, cardiomyopathy, pulmonary edema, adult respiratory distress syndrome, and even death (**Table 1**; Berg et al., 1996; Vikse et al., 2008; Ghulmiyyah and Sibai, 2012). Actually, preeclampsia is responsible for more than 60,000 maternal deaths annually worldwide, placing it as the third cause of maternal mortality after bleeding and embolism (Mongraw-Chaffin et al., 2010; Young et al., 2010). Higher mortality rate was observed when preeclampsia is associated with HELLP (hemolysis, elevated liver enzymes, low platelets), syndrome liver hemorrhage or rupture, acute kidney injury (AKI), oliguria, disseminated intravascular coagulation (DIC), and pulmonary edema (Ghulmiyyah and Sibai, 2012). Preeclampsia is of special relevance in the developing countries, where the maternal mortality is ∼15% compared with 0–1.8% in the developed countries (Ghulmiyyah and Sibai, 2012). This difference is largely attributed to inadequate perinatal care in poor regions of the world, and subsequently missing timely detection of hypertension, generalized or local edema, and proteinuria to detect preeclampsia at early stages.

**Table 1:** Adverse impact of preeclampsia on fetus and mother.

On fetus	On mother
• Growth restriction	• Hypertension
• Preterm delivery	• Future HTN, CVD
• Placental abruption	• Kidney injury
• Respiratory distress	• Chronic kidney disease and risk for ESRD
• Cerebral palsy	• Liver failure
• Retinopathy of prematurity	• Cardiomyopathy
• Necrotizing enterocolitis	• CNS damage and stroke
• Sepsis	• Seizure
• Stillbirth	• Diabetes mellitus
	• Coronary artery disease
	• Pulmonary edema
	• Death


In the last decade, the definition of preeclampsia was revisited as the mechanisms underlying the disease were dramatically evolved. Concerning the former, several leading groups have challenged the half century old classic definition of preeclampsia, namely, *de novo* hypertension, new onset of proteinuria and liver dysfunction after mid pregnancy, motivated by the discovery of additional biomarkers of preeclampsia (Tjoa et al., 2007; Staff et al., 2013; Palomaki et al., 2015; Baltajian et al., 2016). In this context, several studies have suggested to modernize the definition by incorporating key biomarkers of either placental or vascular origins, including placenta growth factor (PlGF) and antiangiogenic factors such as soluble fms-like tyrosine kinase-1 (sFLT1) or soluble endoglin (sENG) in the diagnosis of preeclampsia and the risk for developing the disease and even in predicting the outcome (Tjoa et al., 2007; Staff et al., 2013; March et al., 2015; Palomaki et al., 2015; Sircar et al., 2015; Baltajian et al., 2016). The suggested definition takes into account the impressive advancement in understanding the pathophysiology of preeclampsia and the mechanism-based novel diagnostics and therapeutic options.

In light of the rapid pace in the development of this issue and its clinical relevance, the current review concentrates on recent breakthroughs in diagnosing preeclampsia and the derived therapeutic options, which are currently been tested in advanced clinical trials. The initial results seem encouraging and may break down the old dogma claiming that no intervention has been proved to prevent or delay the onset of preeclampsia and the only effective treatment is delivery.

## Risk Factors for Preeclampsia

Although the mechanisms of preeclampsia are poorly elucidated, there are several predisposing factors that increase the risk for the development of the disease (**Table 2**; Al-Jameil et al., 2014). Among the leading risk factors (yet uncommon) is antiphospholipid antibody syndrome (APLA-S). In addition, numerous epidemiological studies have demonstrated that chronic kidney disease (CKD) significantly increases the risk of preeclampsia, especially lupus (Roberts et al., 1989; Mostello et al., 2002; Clowse et al., 2008; Jim and Karumanchi, 2017). Risk factors for pre-eclampsia include also former preeclampsia, first pregnancy, obesity, pregestational hypertension, older age, and diabetes mellitus (Al-Jameil et al., 2014). It is also more frequent in multifetal pregnancy, where the incidence of preeclampsia is increased in twin compared to singleton pregnancies to 6–31% (McFarlane and Scott, 1976; Coonrod et al., 1995). Despite the association between these risk factors and preeclampsia, the mechanisms whereby these factors increase this risk are largely unknown. However, underlying diseases characterized by imbalance of angiogenetic factors and coagulation may explain why certain populations are at risk. Despite that, in most cases preeclampsia is unpredictable (Jim and Karumanchi, 2017).

**Table 2:** Major predisposing risk factors for the development of preeclampsia.

Risk factor	OR or RR (95% Cl)
Antiphospholipid antibody syndrome	9.7 (4.3–21.7)
Renal disease	7.8 (2.2–28.2)
Prior preeclampsia	7.2 (5.8–8.8)
Systemic lupus erythmatosis	5.7 (2.0–16.2)
Nulliparity	5.4 (2.8–10.3)
HIV+ HAART treatment	5.6 (1.7–18.1)
HIV positive (untreated)	4.9 (2.4–10.1)
Chronic hypertension	3.8 (3.4–4.3)
Diabetes Mellitus	3.6 (2.5–5.0)
Multiple Gestation	3.5 (3.0–4.2)
Strong family history of cardiovascular disease (heart disease or stroke in ≥2 first degree relatives)	3.2 (1.4–7.7)
Obesity	2.5 (1.7–3.7)
Family history of preeclampsia in first degree relative	2.3–2.6 (1.8–3.6)
Advanced maternal age (>40) for multips	1.96 (1.34–2.87)
Advanced maternal age (>40) for nulliparas	1.68 (1.23–2.29)


## Pathogenesis of Preeclampsia

**FIGURE 1:**
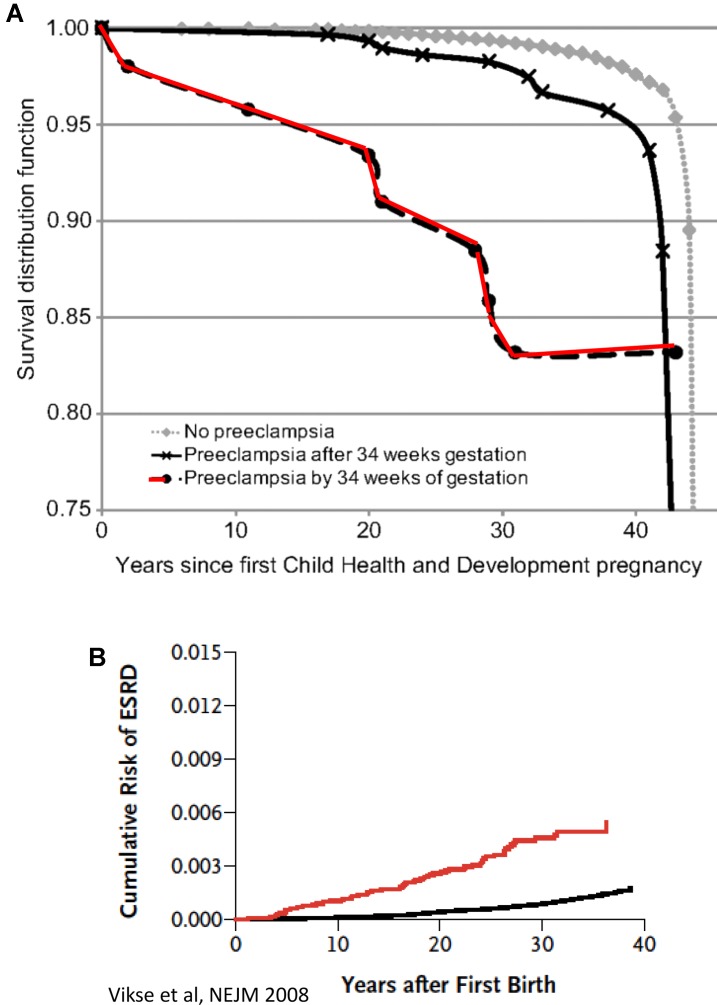
**(A)** CVD death Kaplan–Meier survival according to gestational timing of preeclampsia. Survival analysis is based on 14, 403 pregnant women. A total of 481 had observed preeclampsia, and 266 died from cardiovascular disease (adopted with permission from Mongraw-Chaffin et al., 2010). **(B)** Cumulative risk of end-stage renal disease (ESRD) after first preeclampsia (adopted with permission from Vikse et al., 2008).

In the last decade, our understanding of the pathogenesis of preeclampsia has progressively advanced (Phipps et al., 2016). Therefore, in this section we will focus on the most recent concepts in the pathogenesis of the disease, especially the involvement of angiogenic factors. It is obvious today that preeclampsia is a systemic disease characterized by generalized endothelial damage (Roberts et al., 1989), thus negatively affecting almost all organs of preeclamptic women, including the potential to affect future cardiovascular and renal diseases even decades after the disease occurrence (**Figure 1**; Berg et al., 1996; Vikse et al., 2008). In this context, a comprehensive prospective study revealed that preeclampsia was independently associated with cardiovascular disease death (mutually adjusted hazard ratio: 2.14 [95% CI: 1.29–3.57]) (Mongraw-Chaffin et al., 2010). The situation was even grimmer in women who experienced preeclampsia by 34 weeks of gestation (HR, 9.54; 95% CI, 4.50–20.25) (Mongraw-Chaffin et al., 2010). The high mortality rate could be explained by the findings that early-onset preeclampsia conferred a substantially higher risk of cardiovascular, respiratory, CNS, renal, hepatic, and other morbidity and was evident by end target damage (Lisonkova et al., 2014). Collectively, these findings suggest that the risk of morbidity/mortality among preeclamptic women is related to the severity the disease and gestational age at onset, namely early (<34 weeks) or late (>34 weeks). However, it should be emphasized that if the mother has a genetic predisposition to cardiovascular disease, then it is this rather than pre-eclampsia *per se* that causes the increased morbidity in later life as outlined above. Therefore, additional studies are needed to distinguish between the contribution of preeclampsia itself and the genetics to the high prevalence of cardiovascular morbidity and mortality among preeclamptic women.

It is now appreciated that early- and late-onset pre-eclampsia have different pathophysiologies, thus advancing our understanding of the syndrome. In early-onset, also referred to as placental pre-eclampsia, there is clear evidence of reduced maternal spiral artery conversion in early pregnancy. This is associated with placental malperfusion, and gross and molecular pathology of the placental tissues. Oxidative stress of the placenta causes increased secretion of sFLT-1 and reduced PlGF, reflecting the biomarker patterns. In late-onset pre-eclampsia, called also maternal preeclampsia, there is little evidence of reduced arterial conversion and placental perfusion is maintained or even increased (Sohlberg et al., 2014). Thus, there is only minimal placental stress (Yung et al., 2005) so that sFLT and placental growth factor (PlGF) secretion by the placenta are close to the normal range. These cases, which represent nearly 80% of pre-eclampsia, are now thought to be due to a genetic maternal pre-disposition to cardiovascular disease, which manifests as pre-eclampsia during the stress-test of pregnancy.

The pathology early-onset preeclampsia starts with abnormal formation of blood vessels in the maternal uterine spiral arteries. During normal pregnancy, major adaptive changes take place including spiral artery remodeling in the pregnant uterus aimed at decreasing maternal blood vessel resistance and subsequently increasing unteroplacental perfusion (Lyall, 2005). However, mathematical modeling shows that the remodeling has relatively little impact on uteroplacental perfusion, and is more concerned with reducing the velocity of inflow and ensuring constancy of blood flow (Burton et al., 2009).

**FIGURE 2:**
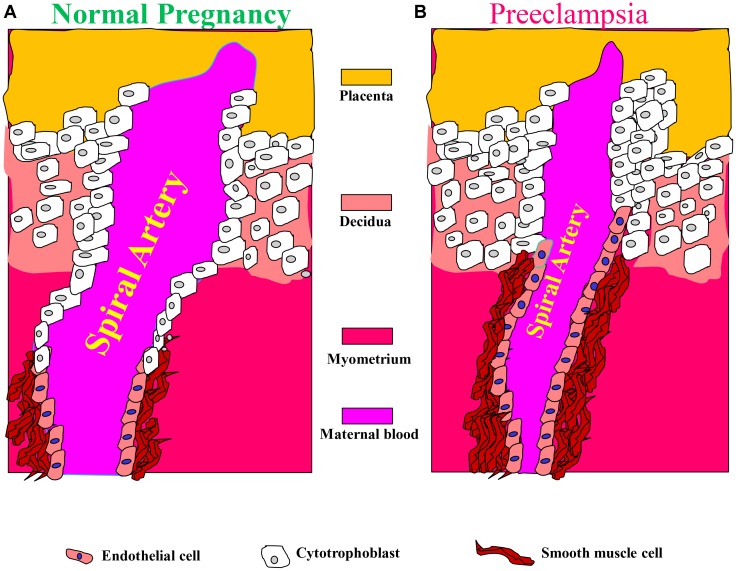
Aberrant placentation and angiogenesis in preeclampsia. In normal pregnancy, cytotrophoblasts of fetal origin invade the maternal spiral arteries and replace endothelial cell layer. This action converts the spiral arteries from narrow highly resistant vessels to high-caliber capacitance vessels, which are capable of providing sufficient blood and nutrition supply to the fetus. During the process of vascular invasion, the cytotrophoblasts differentiate from an epithelial to an endothelial phenotype, a process referred to as pseudovasculogenesis, or vascular mimicry (**Right**). In preeclampsia, cytotrophoblasts fails to acquire invasive endothelial phenotype features, thus the invasion of the spiral arteries is inadequate leaving them narrow and highly resistant (**Left**). Modified with permission from Lam et al. (2005) and Powe et al. (2011).

These alterations in spiral arteries, namely high-capacitance low-pressure flow to the placenta, are essential for fetal nutrition. Spiral artery remodeling is achieved through invasion of trophoblasts and disappearance of the smooth muscle in the blood vessel wall (Kaufmann et al., 2003; Lyall, 2005; Osol and Mandala, 2009). Using mouse model revealed that this process involves the full circumference of the vessel in its segment entering the placenta from the mesometrial triangle, so called, the central canal. The deeper parts of the spiral artery within the mesometrial triangle and even beyond it, as deep as the mesometrium, are only partially remodeled and retain the muscular wall in part of their circumference (Geusens et al., 2008; Skarzinski et al., 2009; **Figure 2**). In order to achieve spiral remodeling during normal pregnancy, many molecules including vasoactive substances, growth factors, adhesion molecules and proteases are secreted by the placenta and the vasculature (Brosens et al., 1972; Norwitz et al., 2001; Kaufmann et al., 2003; Lyall, 2005; Pijnenborg et al., 2006). Among the most famous representative substances in this context are vascular endothelial growth factor (VEGF), sFlt1, PlGF, and endoglin (Takimoto et al., 1996; Maynard et al., 2003; Levine et al., 2004; Li et al., 2005; Venkatesha et al., 2006; Kanasaki et al., 2008; Zhou et al., 2008). Furthermore, interactions with the maternal immune cells, especially uterine natural killer cells and their corresponding human leukocyte antigen-C (HLA-C) ligands on the invading trophoblast, are important for release of proteases and remodeling (Moffett et al., 2015). Interference with their central role in creating efficient uteroplacental interface and cardiovascular and renal adaptations during pregnancy contributes to preeclampsia as elaborated below. It is widely accepted that abrupt remodeling of the uterine spiral arteries plays a key role in the pathogenesis of early onset preeclampsia (Brosens et al., 1972; Norwitz et al., 2001; Kaufmann et al., 2003; Red-Horse et al., 2004; Pijnenborg et al., 2006), yet there is no evidence that they are involved in arterial remodeling of the spiral arteries.

### Angiogenic Factors

**FIGURE 3:**
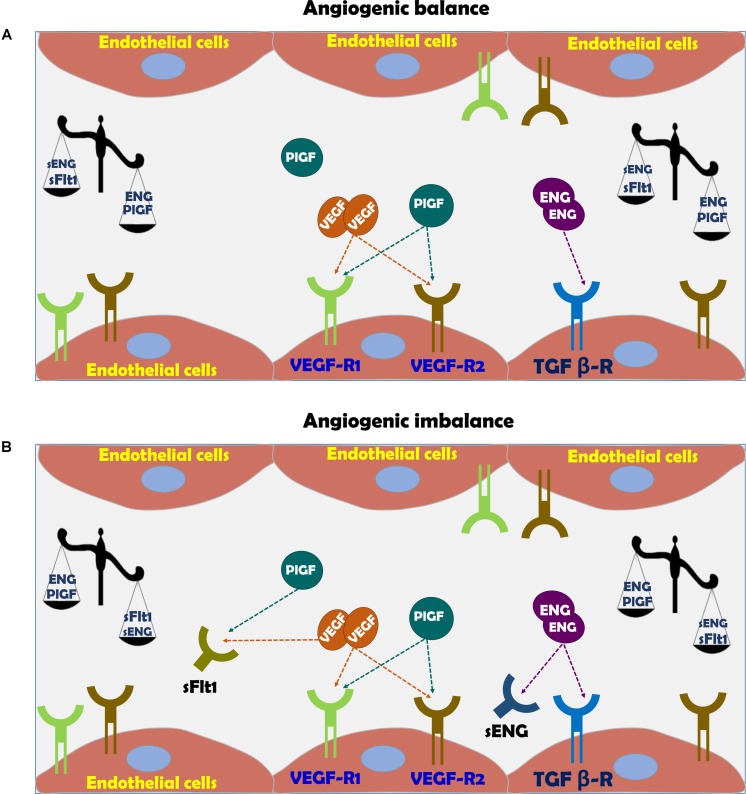
**(A)** Vascular function in normal pregnancy and **(B)** Vascular dysfunction in preeclampsia. In normal pregnancy there is angiogenic balance as evident by VEGF, PlGF, and TGF-β levels, which are necessary for normal blood vessel formation and endothelial function via eNOS activation and subsequent nitric oxide generation. The action of these factors is hampered in preeclampsia due to angiogenic imbalance, where soluble sFlt-1 and sEng act as scavengers for native VEGF, PlGF, and TGF-β, thus inducing endothelial dysfunction.

As mentioned above, insufficient spiral artery remodeling due to superficial invasion of trophoblasts is the basis for the development of early-, but not late-onset cases of preeclampsia (Brosens et al., 1972; Norwitz et al., 2001; Kaufmann et al., 2003; Red-Horse et al., 2004; Pijnenborg et al., 2006). Perturbations in the generation of normal uteroplacental interface results in ischemic placenta and oxidative stress which stimulates the release of prohypertensive and anti-angiogenic factors (such as sFlt-1) (Cindrova-Davies, 2009). Moreover, sFlt-1 sensitizes the endothelial cells of the maternal circulation to pro-inflammatory cytokines such as tumor necrosis factor-α (TNF-α) (Cindrova-Davies et al., 2011), causing generalized endothelial dysfunction and subsequently multisystem damage (**Figure 3**; Roberts et al., 1989; Llurba et al., 2015; Verdonk et al., 2015).

#### Placenta Growth Factor

Among these substances are VEGF sFlt1, PlGF, sENG, and endothelin (ET-1) (**Figure 3**; Llurba et al., 2015; Verdonk et al., 2015). Therefore, sFlt-1, PlGF and endoglin are extensively assessed as potential biomarkers for the diagnosis of preeclampsia (Venkatesha et al., 2006; Staff et al., 2013). While PlGF is proangiogenic, sFlt-1 is antiangiogenic factor (Ahmed, 2011). PlGF is expressed by the placenta, especially the syncytiotrophoblast (Maglione et al., 1991), but also by the endothelium (Staff et al., 2013). PlGF is a prominent angiogenic player in the development of the placental vascular system (Iwasaki et al., 2011; De Falco, 2012). During normal pregnancy, PlGF can be detected in the maternal circulation from 8 weeks gestation, reaching a maximal concentration toward the end of second trimester and declining thereafter until delivery (Taylor et al., 2003). In line with its proangiogenic function, reduced levels of PlGF were found in preeclampsia (George and Granger, 2010; Staff et al., 2013; Kar, 2014). It is noteworthy that infusion of recombinant human PlGF via intraperitoneal osmotic minipumps abolished the development of hypertension in experimental preeclampsia model (Spradley et al., 2016).

#### Soluble fms-Like Tyrosine Kinase-1

Vascular endothelial growth factor is critical for vascular homeostasis and activates both VEGF receptor-1 (VEGFR-1) and VEGFR-2 coupled to endothelial nitric oxide synthase (eNOS) required for angiogenesis (**Figure 3**; Ferrara, 2004; Ahmad et al., 2006; Sison et al., 2010; Bertuccio et al., 2011; Veron et al., 2012). The importance of VEGF for the maintenance of normal endothelial function and development of placental vasculature is derived from the consequences of impairment of VEGF activity due to certain drugs or elevation of sFlt-1 (Ahmed, 1997; Kabbinavar et al., 2003; Sison et al., 2010; Bertuccio et al., 2011; Veron et al., 2012). In this context, anti- VEGF therapy with Avastin display preeclampsia-like symptoms, namely hypertension and proteinuria (Kabbinavar et al., 2003; Eremina et al., 2008; Muller-Deile and Schiffer, 2011; Hayman et al., 2014). sFlt-1 is a splice variant of VEGF receptor fms-like tyrosine kinase 1 (Maynard et al., 2003). sFlt-1 acts as a potent scavenger of VEGF- and PlGF, thus preventing their interaction with endothelial receptors on the cell surface and subsequently induces endothelial dysfunction (**Figure 3**; Kendall and Thomas, 1993; Levine et al., 2004). The elevation of sFlt-1 is due to overexpression of sFlt-1 mRNA as was demonstrated in *in vivo* and *in vitro* models of human placental hypoxia mediated by hypoxia inducible factor 1 (HIF-1) (Nevo et al., 2006; Onda et al., 2017). Support for its pro preeclamptic role came from experimental studies, where administration of adenoviral enhanced overexpression of sFlt-1 into pregnant rats or mice, induced clinical manifestations of preeclampsia, including profound elevation of blood pressure (BP), albuminuria, and renal histologic changes such as endotheliosis and fibrin deposition within the enlarged glomeruli (Gartner et al., 1998; Maynard et al., 2003; Onda et al., 2017). At the mechanistic level, sFlt-1 indirectly prevents the production of VEGF-induced NO, resulting in enhanced generation of reactive oxygen species and exaggerated vasoconstriction (Ahmad and Ahmed, 2004; Burke et al., 2016).

In clinical setting, sFlt-1 levels were found to be elevated as early as 5 weeks before the diagnosis of preeclampsia and directly correlate with disease severity (Levine et al., 2004; Kar, 2014). Furthermore, support for sFlt-1 role in the pathogenesis of preeclampsia is derived from experimental and clinical studies involving sFlt-1 elimination (Ahmad and Ahmed, 2004; Thadhani et al., 2016; Jim and Karumanchi, 2017). Specifically, sFlt-1 removal by dextran sulfate apheresis in humans reduced proteinuria and prolonged pregnancy (Thadhani et al., 2016).

#### Endoglin

Endoglin (ENG) is a type I membrane glycoprotein localized to the cell membrane where it constitutes the transmembrane co-receptor for TGF beta receptor complex (TGF-β1 and TGF-β3) (Gregory et al., 2014). ENG is expressed by endothelial cells and monocytes, especially during neoangiogenesis and embryogenesis (Gregory et al., 2014). Concerning the latter, the human placenta, especially syncytiotrophoblast is an important source of ENG (Gougos and Letarte, 1990). The primary roles of ENG include angiogenesis, endothelial cell differentiation and regulation of vascular tone through eNOS (Ahmad and Ahmed, 2004). Proteolytic cleavage of the extracellular domain of endoglin, generates sEng that presumably functions as limiting factor for the activity of TGF-β and the coupled eNOS (**Figure 3**; Qu et al., 1998; Bourdeau et al., 1999). Since TGF-β acts as anti-inflammatory and vasodilator growth factor, its elimination by sEng leads to endothelial dysfunction characterized by vasoconstriction, overexpression of adhesion molecules and reduced T cells characterizing preeclamptic women (Matsubara et al., 2000; Ahmed, 2011). By using experimental model of preeclampsia, it was shown that sEng and sFlt-1 act synergistically to induce endothelial dysfunction especially the severe variant of the disease, namely HELLP syndrome (Santner-Nanan et al., 2009). Similarly, circulating sEng was found to be high in preeclamptic women even prior to the disease manifestations correlating with disease severity and falls after delivery (Levine et al., 2004; Venkatesha et al., 2006), making it a reliable predictor of patients destined to develop severe early-onset preeclampsia (Robinson and Johnson, 2007). The regulators of sEng release are largely unknown, however like sFlt-1, it was reported that both cytokines (Zhou et al., 2010), and autoantibodies to angiotensin II AT-1 receptors stimulate (Cudmore et al., 2007) and heme oxygenase-1 (HO-1) inhibits its release (see later) (Zhou et al., 2010).

#### Other Vasoactive Substances

One of the major features of preeclampsia is generalized vasoconstriction and reduced plasma volume, assumedly due to endothelial activation even weeks before clear evidence of the disease (Roberts et al., 1989; Roberts and Lain, 2002). Endothelial dysfunction is characterized by reduced blood flow to virtually all organs in preeclamptic women due to vasoconstriction. The latter is partially attributed to imbalance in neurohormonal systems, including activation of the sympathetic nervous system and renin angiotensin aldosterone system (RAAS) as well as endothelin (ET-1) (Gant et al., 1973; Roberts and Lain, 2002). On the other hand, endothelium-dependent vasodilation (PGs, VEGF, TGF-β, and NO system) is also attenuated in preeclamptic patients (Fischer et al., 2000; Yoshida et al., 2000), secondary to oxidative stress which is known to provoke endothelial dysfunction (Roberts et al., 1989; McKinney et al., 2000). Partial restoration of the balance (even for a short while) by water immersion of preeclamptic women increased cardiac output and reduced systemic vascular resistance (SVR), yet to a lower extent than normal pregnant women (Elvan-Taspinar et al., 2006). Yet, the therapeutic potential for water immersion in preeclampsia appears to be limited (Elvan-Taspinar et al., 2006). Several studies have reported elevated ET-1 levels in preeclampsia and some of them demonstrated a positive correlation between ET-1 and the severity of symptoms (Taylor et al., 1990; Mastrogiannis et al., 1991; Benigni et al., 1992; Granger et al., 2006; George and Granger, 2011, 2012; George et al., 2012). The cadence of ET-1 as mediator of many preeclampsia manifestations is appealing in light of its potent vasoconstrictory, inflammatory and proteinuric properties (Davenport et al., 2016; Saleh et al., 2016; Bakrania et al., 2017). Support for this notion is derived from animal models of preeclampsia, where it has been shown that endothelin receptor blockers prevent the development of the disease (Saleh et al., 2016; Bakrania et al., 2017).

Finally, HO-1 plays an anti-inflammatory and inhibitory role on sFlt-1 and sEng release via its metabolite carbon monoxide (CO) (Ahmed, 2011). In line with HO-1 involvement in the pathogenesis of preeclampsia, women with the disease exhale less CO than women with normal pregnancies and HO-1 expression decreases as the severity of preeclampsia increases (Ahmed, 2011). The downregulation of HO-1 aggravates the inflammatory aspect of preeclampsia, and deprives of the body from important anti stress and anti-oxidant defense mechanism (Ahmed, 2011).

#### Diagnosis of Preeclampsia

For more than half century, the clinical syndrome of preeclampsia is defined as *de novo* hypertension and new onset of proteinuria after mid pregnancy (ACOG practice bulletin, 2002). Hypertension is diagnosed when it is greater than 140 mmHg systolic or 90 mmHg diastolic at two separate times, more than 4 h apart in a woman after 20 weeks of gestation (Duley, 2003). In addition, proteinuria of >300 mg/day is milestone for the diagnosis of preeclampsia (Staff et al., 2013). However, in the last decade this concept has been challenged in light of the fact that the disease develops long time prior to its keen manifestations (Staff et al., 2013). Actually, early clinical signs of preeclampsia may be absent or unremarkable, and the reliability of these two hallmarks (hypertension and proteinuria) as gold standard is compromised, especially if the pregnant women suffer from predisposing conditions, such as chronic hypertension and CKD (Sibai and Stella, 2009). Therefore, the search for more sensitive and early biomarker of the disease continued all the time and is more zeal in the last decade. This issue is of great importance since early diagnosis of preeclampsia may be the first step in the journey for the development of effective treatment, especially if the biomarkers are of mechanistic relevance. In this context, new biomarkers were derived from the recent unprecedented advances in our understanding of the pathogenic mechanisms underlying preeclampsia (Ahmed, 2011; Staff et al., 2013; Phipps et al., 2016; Jim and Karumanchi, 2017). Specifically, it is now obvious that angiogenic imbalance, as reflected by elevated levels of sFlt-1, sEng, and ET-1 along decreased PlGF concentrations in the maternal circulation (Ahmed, 2011; Staff et al., 2013; Phipps et al., 2016; Saleh et al., 2016; Jim and Karumanchi, 2017), is the link between this syndrome and the malperfused placenta characterizing the early-onset pre-eclampsia, and the maternal genetic predisposition, as in the late-onset form. Therefore, sFlt-1, sEng, and PlGF are mounting biomarkers for the diagnosis of preeclampsia (Ahmed, 2011; Staff et al., 2013; Phipps et al., 2016; Jim and Karumanchi, 2017). Besides their diagnostic features, these biomarkers were found to possess prognostic features. For instance, Rana et al. (2013) showed that high ratio of sFlt to PlGF in preeclamptic women is associated with worse maternal and fetal outcomes compared with women with a lower ratio.

In a prospective multicenter observational study, Zeisler et al. (2016) examined whether ratio of serum sFlt-1 to PlGF predicts the absence or presence of preeclampsia in the short term in women with singleton pregnancies in whom preeclampsia was suspected (24 weeks 0 days to 36 weeks 6 days of gestation). These authors have shown that an sFlt-1-to-PlGF ratio of 38 or lower drawn at 24–37 weeks of gestation can reliably predict the absence of preeclampsia and fetal adverse outcomes within 1 week, with negative predictive values of 99.3 and 99.5%, respectively. Similarly, Sovio et al. (2017) who determined sFlt-1:PlGF ratio at 20, 28, and ≈36 weeks of gestational age in 4,099 women recruited to Pregnancy Outcome Prediction. At 28 gestational week, a sFlt-1:PlGF ratio >38 had a positive predictive value of 32% for preeclampsia and preterm birth. At 36 weeks, a sFlt-1:PlGF ratio >38 had a predictive value for severe preeclampsia of 20% in high-risk women and 6.4% in low-risk women. When sFlt-1:PlGF ratio was >110 it has predictive value of 30% for severe preeclampsia. Among low-risk women at 36 weeks, a sFlt-1:PlGF ratio ≤38 had a negative predictive value for severe preeclampsia of 99.2%. Collectively, the sFlt-1:PlGF ratio provided clinically useful prediction of the risk of the most important manifestations of preeclampsia, confirming the pioneer findings by Levine et al. (2004) and providing rational for the use of angiogenic biomarkers to stratify women at high risk for preeclampsia.

In similarity with sFlt-1, serum concentrations of sEng are elevated in preeclamptic women (Chen, 2009), as compared with stable levels throughout normal pregnancy. A positive correlation between the elevated serum levels of sEng and the severity of pre-eclampsia has been demonstrated (Chen, 2009). Noteworthy, serum sEng have been shown to be significantly increased before the onset of disease. Specifically, sEng levels increased as early as 9–11 weeks in pregnant women at risk for preeclampsia and by12–14 weeks in women with term preeclampsia (Levine et al., 2004). Thus, sEng could be used to predict preeclampsia at 11–13 week gestation (Akolekar et al., 2011) with precaution since high levels of sEng are detected also in other gestational disorders such as small gestational age, thus limiting its specificity. Therefore, the pattern of changes in the ratio of different combinations of PlGF/sEng; sflt-1 + sEng)/PlGF, at 13 weeks and around 20 weeks, is more informative than the individual biomarkers at single time-point screening (Levine et al., 2004; Romero et al., 2008; Rana et al., 2013).

#### Kidney Placenta Crosstalk

##### The kidney in normal pregnancy

There is an important crosstalk between the placenta and the kidney during normal pregnancy, as evident by adaptive anatomic and physiologic renal changes. The latter include an increase in renal size by 30% and length by 1–1.5 cm mainly due to the increased renal blood flow (RBF) as early as the first 4 weeks of pregnancy (Hussein and Lafayette, 2014). The collecting systems of both kidneys are normally dilated and are more pronounced on the right, therefore “Physiological hydronephrosis” can occur in late pregnancy. Moreover, reduction in BP secondary to generalized peripheral vasodilation due to the reduced SVR, is probably due to the increased resistance to angiotensin II (Gant et al., 1973). Likewise, imbalance between the vasodilatory prostacyclin and relaxin and vasoconstrictive thromboxane in favor of the first, and activation of nitric oxide (NO), a potent vasodilator that mediates endothelium dependent relaxation (Hussein and Lafayette, 2014), may contribute to this phenomenon. At the renal level, there is an increase in glomerular filtration rate (GFR) secondary to increased RBF by ∼35–50% (Hussein and Lafayette, 2014). For this reason, normal pregnancy is accompanied by low serum creatinine, urea, sodium, uric acid levels and increased urinary protein excretion up to 300 mg/d (Hussein and Lafayette, 2014). The renal vasodilation is responsible also for the activation of the RAAS. In fact, despite the elevated levels of renin and aldosterone in pregnant woman, both the BP and SVR are reduced (Gant et al., 1973).

##### The kidney in preeclampsia

**FIGURE 4:**
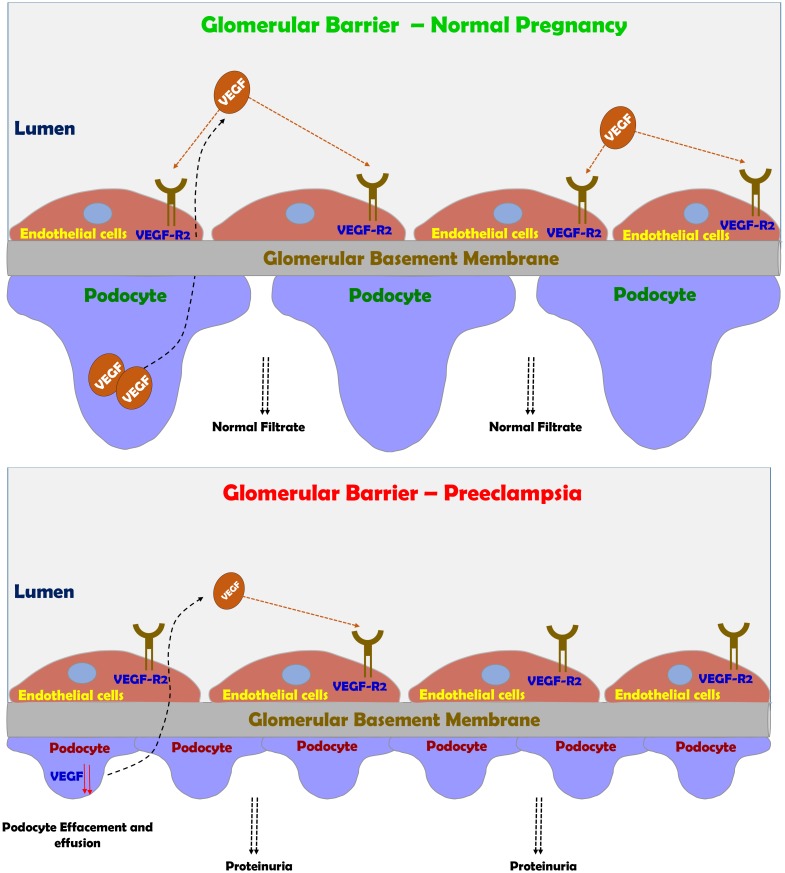
Preeclampsia-induced renal dysfunction. Under normal condition, VEGF plays a central role in maintaining the integrity of glomerular barrier as evident by healthy podocytes with normal foot process and slit diaphragm proteins. Under preeclamptic condition, angiogenic imbalance develops mainly due to sFlt-1 and sEng, leading to podocyte and endothelial damage in the glomerular filtration barrier and subsequently to the development of proteinuria.

The above-mentioned changes are partially realized in complicated pregnancy such as preeclampsia, where pathologic changes in both the placenta and the kidneys take place. One of the most vulnerable organ to miss adaptive changes during preeclampsia is the kidney, where glomerular endotheliosis and proteinuria develop (Spargo et al., 1959; Gartner et al., 1998; Craici et al., 2013). The hallmark characteristic renal pathologic lesion of preeclampsia “glomerular endotheliosis” is characterized by an enlarged bloodless glomerulus with obliteration of the capillary lumen, but usually not accompanied by prominent capillary thrombi (**Figure 4**; Spargo et al., 1959; Hussein and Lafayette, 2014). Initially, endothelial cell swelling and disruption of their fenestrae were thought to be the cause of proteinuria seen in preeclampsia (Hussein and Lafayette, 2014). However, there is increasing evidence that damage to the podocytes, the visceral epithelial glomerular cell, is largely responsible for the proteinuria (**Figure 4**). In this context podocyturia (loss of podocytes in the urine), along shedding of slit diaphragm proteins such as nephrin, podocin, synaptopodin and podocalyxin were noticed in preeclampsia, and even precede the typical clinical features of preeclampsia by several weeks (Garovic et al., 2007a,b, 2013; Aita et al., 2009; Zhao et al., 2009, 2011; Facca et al., 2012; Jim et al., 2012; Kelder et al., 2012; Wang et al., 2012; Chen et al., 2013; Son et al., 2013). It should be emphasized that these slit diaphragm proteins play a key role in maintaining the integrity of the glomerular barrier (Kestila et al., 1998).

The end result of this disruption of the glomerular filtration barrier and podocyte detachment is proteinuria (**Figure 4**; Spargo et al., 1959; Craici et al., 2013). In fact, there is a positive correlation between the grade of podocyturia and the severity of proteinuria. One of the main mediators of the adverse renal consequences of preeclampsia is ET-1 (Taylor et al., 1990; Mastrogiannis et al., 1991; Benigni et al., 1992; Granger et al., 2006; George and Granger, 2011, 2012; George et al., 2012; Verdonk et al., 2015; Davenport et al., 2016; Saleh et al., 2016; Bakrania et al., 2017). Support for this notion is derived from the observation that pre-eclamptic sera are not directly toxic to cultured podocytes, but if the glomerular capillary endothelium is exposed to sera from pre-eclamptic women it produces podocytes damage via ETA receptor subtype (Collino et al., 2008). Addition keen evidence for the involvement of ET-1 system in the pathogenesis of renal dysfunction characterizing preeclampsia came from the observation that podocyte damage and shedding can be prevented by ETA blockers (George and Granger, 2011; Verdonk et al., 2015; Bakrania et al., 2017). However, in the last decade, there is growing evidence that imbalance between the proangiogenic and anti angiogenic factors plays a key role in podocyte injury (Genbacev et al., 1997; Fulton et al., 1999; Robertson et al., 2003; Venkatesha et al., 2006; Baelde et al., 2007; Sison et al., 2010; Bertuccio et al., 2011; Veron et al., 2012). This concept is supported by the observation that bevacizumab, an anti-VEGF antibody used to treat patients with various types of cancer or diabetic proliferative retinopathy causes hypertension and proteinuria mimicking the effect of sFlt-1 (Eremina et al., 2008; Muller-Deile and Schiffer, 2011; Hayman et al., 2014). Interestingly, the renal findings in patients who were treated with bevacizumab including endotheliosis, thrombotic microangiopathy, and podocytes shedding, are similar to those found in preeclamptic state (Eremina et al., 2008; Muller-Deile and Schiffer, 2011; Hayman et al., 2014). Kidney damage during preeclampsia as evident by endothelial and podocytes’ loss contributes to the increased risk of later hypertension, CKD, ischemic heart disease, stroke, persistent proteinuria and finally ends stage renal disease (ESRD) (LaMarca B.D. et al., 2008; Vikse et al., 2008, 2012; McDonald et al., 2010; Kattah et al., 2013; Wang et al., 2013).

As mentioned above, superficial placental implantation due to abnormal angiogenesis is the early driving event for the development of preeclampsia. The imbalance between the pro-angiogenic VEGF and PlGF, and the antiangiogenic sFlt-1 and sEng plays a central role in the pathogenesis of placental hypoxia, as both VEGF and PlGF are essential for fetal and placental angiogenesiss (Lam et al., 2005; Sison et al., 2010; Bertuccio et al., 2011; Powe et al., 2011; Rana et al., 2012; Veron et al., 2012). Excessive production of antiangiogenic sFlt-1 and sEng reduces the bioavailability of free pro-angiogenic PlGF and VEGF, by binding and neutralizes VEGF and PlGF, thus reducing the availability of free VEGF for fetal and placental angiogenesis. In comparison, the sEng is implicated in neutralizing TGF- β, an anti-inflammatory growth factor (Roberts et al., 1989) that activates eNOS (Phipps et al., 2016). This imbalance leads to systemic endothelial dysfunction, including in the kidney (Kaufmann et al., 2003; Venkatesha et al., 2006), where disruption of slit diaphragm was reported (Garovic et al., 2007a; Henao et al., 2008; Zhao et al., 2009), as VEGF is essential for the maintenance glomerular barrier (Baelde et al., 2007). Support for the adverse effect of preeclampsia on glomerular barrier was reported by Henao et al. (2008). These authors demonstrated that when a human podocyte cell line was stimulated with serum from women with preeclampsia, disruption of CD2AP, podocin and actin were observed, but not when sera from normal pregnancy was added. Furthermore, the mean resistance value of podocytes cultured with serum from women with preeclampsia was significantly lower than podocytes cultured with serum from controls. This effect is mediated by ET-1 release by endothelial glomerular cells as preeclamptic sera induce nephrin shedding from podocytes (Romero et al., 2008). In this context, Elevated levels of ET-1, autoantibodies to the angiotensin II type I receptor, tumor necrosis factor α (TNFα) and interleukin-6 (IL-6) are also elevated in pre-eclampsia (Taylor et al., 1990; Mastrogiannis et al., 1991; Benigni et al., 1992; Granger et al., 2006; LaMarca B. et al., 2008; LaMarca B.D. et al., 2008; George and Granger, 2011, 2012; George et al., 2012).

Preeclampsia is commonly (but not always) accompanied by new onset proteinuria (>300 mg/d) or worsening proteinuria diagnosed after 20 weeks of pregnancy and generalized edema. The latter is mainly due to primary renal retention of salt and water despite the suppression of RAAS during preeclampsia due to vasoconstriction, in contrast to its upregulation in normal pregnancy, which is characterized by vasodilation (Schrier, 1988). Thus, the edema-accompanied preeclampsia resembles the “over-fill” edematous clinical settings. Another abnormal laboratory tests include elevated levels of creatinine, urea, uric acid levels along hypocalciuria, decreased urate excretion, and proteinuria.

#### Novel Mechanisms Based Therapeutics

**FIGURE 5:**
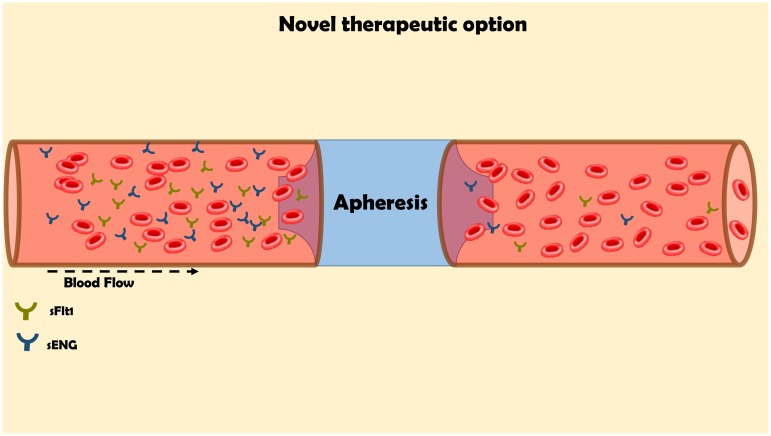
Innovative approach for the elimination of the elevated levels of sFlt-1 by apheresis for the management of preeclampsia. The maternal blood flow through columns, which contains antibodies against sFlt1 in order to remove it from the circulation.

Despite the rapid progress in understanding the mechanisms underlying the pathogenesis of preeclampsia, the treatment options remained very limited, except for early delivery. The current treatment options such as low Na^+^ diet, diuretics, Ca^++^ supplementation, Vitamin C and E were ineffective in most cases (Jim and Karumanchi, 2017). Aspirin moderately reduced the incidence of preterm preeclampsia in high-risk patients when given the drug at 11–14 weeks of gestation until 36 weeks (Rolnik et al., 2017). Therefore, there is unmet need for novel therapies to treat preeclampsia. Fortunately, as a token for unraveling the role of soluble vascular factors in preeclampsia, several new therapeutics have been developed that target implicated circulating angiogenic factors, including sFlt-1 (**Figure 5**; Sircar et al., 2015; Jim and Karumanchi, 2017). Specifically, these strategies rely on correcting the angiogenic balance, either by promoting proangiogenic factors or by blocking those of antiangiogenic properties. As outlined above, sFlt 1 is involved in the hemodynamic and pathophysiologic changes characterizing preeclampsia such as hypertension, renal dysfunction and shallow placentation (as the case in early-onset preeclampsia). Thus, elimination or reduction of the circulating levels of this deleterious anti-angiogenic factor below critical levels is supposed to ameliorate the angiogenic imbalance. Restoring angiogenic balance eventually improves the clinical signs of preeclampsia as has been confirmed in clinical and experimental models of the disease (Bergmann et al., 2010). In line with this assumption, an early study in five women with severe, early onset preeclampsia has demonstrated that negatively charged dextran sulfate cellulose column apheresis significantly decreased the plasma levels of sFlt-1 and attenuate the deleterious manifestations of the disease, including BP and proteinuria (Thadhani et al., 2011). Interestingly, pregnancy was prolonged by 15–23 days in these women without substantial side effects on the mother or fetus. In agreement with these results, a recent study has demonstrated that sFlt-1 removal by more efficient extracorporeal removal approach in 11 women who suffered from severe, early preeclampsia reduced proteinuria and prolonged pregnancy by 2–21 days depending on the number of courses underwent by the women, without causing major adverse maternal or fetal consequences (**Figure 5**; Thadhani et al., 2016). Additional approach for the reduction of sFlt-1 applied proton pump inhibitors (PPIs) in experimental model of the disease, namely placental sFlt-1 transgenic mice and *in vitro* human primary placental tissue and HUVEC (Onda et al., 2017). Proton pump inhibitors (PPIs) decrease sFlt-1 and sEng secretion, attenuate endothelial dysfunction, dilate blood vessels, decrease BP, and exert antioxidant and anti-inflammatory effects. The authors concluded that PPIs have therapeutic potential for preeclampsia and other diseases characterized by endothelial dysfunction (Onda et al., 2017). At the clinical level, PPI used by pregnant women (430 in number) was associated with decrease in sFlt-1 (Saleh et al., 2017). Moreover, their plasma endoglin and ET-1 levels were lower while sFlt-1 levels correlated positively with both. These findings suggest that PPI may bear therapeutic potential for preeclampsia, although prospective trials are still warranted.

In agreement with their physiological role, replenishing the circulatory levels of VEGF or PlGF exerted beneficial effects in experimental preeclampsia. Specifically, recombinant human PlGF supplementation restores the imbalance and abolished hypertension and GFR impairment in a rat preeclampsia model induced by reduced uterine perfusion pressure (RUPP) (Spradley et al., 2016). Since 3-hydroxy-3-methyl-glutaryl-coenzyme A reductase inhibitors (statins) have been shown to ameliorate the signs of experimental preeclampsia via upregulation of PlGF expression (Kumasawa et al., 2011), their efficacy and safety for prevention of preeclampsia are currently being tested in two clinical trials (Ahmed, 2011; Costantine and Cleary, 2013). Additional alternative to these approaches is administration of VEGF 121, which was shown to alleviate symptoms of preeclampsia including hypertension and renal injury (proteinuria, glomerular endotheliosis) in experimental model of the disease (Li et al., 2007). Co-administration of adenovirus and VEGF in an sFlt-1-induced model of preeclampsia rescued endothelial dysfunction along reduction of free circulatory sFlt-1 by ∼70% (Bergmann et al., 2010). Similarly, chronic infusion of VEGF121 via osmotic minipumps during late gestation reduced sFlt-1, restores GFR and endothelial function, and reduces high BP in experimental model of placental ischemia (Li et al., 2007). Interestingly, in all the above-mentioned studies administration of VEGF to sFlt-1 transgenic animals caused reduction of sFlt-1 along improvement of endothelial function (Bergmann et al., 2010). Although these results suggest that VEGF121 may be a candidate molecule for management of preeclampsia and its related complications, it should be emphasized that such approach may increase the fetal weight as the case in diabetic women, and may cause undesirable side effects such as edema due to its unselective binding to both VEGFR-1 and VEGFR-2.

Although, experimental studies in animal models of preeclampsia have shown that endothelin receptor blockers prevent the development of the disease (Saleh et al., 2016; Bakrania et al., 2017), clinical trials are still needed to validate these promising findings. Moreover, it should be emphasized that rebalancing the angiogenic ratios will improve the peripheral manifestations of the syndrome, but do not impact the underlying pathology of the early-onset cases, such as the acute atherotic changes in the spiral arteries. Hence, there a is danger of increasing stillbirths through prolonging a pregnancy in which uteroplacental perfusion is impaired.

## Summary

Preeclampsia is a multifactorial clinical state that adversely affects almost all vital organs of pregnant women. After a half century of stumbling in understanding the molecular basis of the disease, the last decade has witnessed great advancement in the research of preeclampsia as evident by the discovery of wide battery of novel biomarkers that allow early diagnosis of the disease and prediction of the outcome.

In early-onset, pre-eclampsia there is clear evidence of reduced maternal spiral artery conversion in early pregnancy due to deficient trophoblast invasion and arterial remodeling, resulting in aberrant maternal–fetal interactions during early pregnancy and placental malperfusion. Oxidative stress of the placenta causes the increased secretion of sFLT-1 and reduced PlGF, and so explains the biomarker patterns. The abnormal angiogenic ratios are subsequent to the impaired placentation, and not the cause of it, as there is no evidence that sFLT affects trophoblast invasion. In contrast, in late-onset pre-eclampsia there is slight reduction in arterial conversion and the placental perfusion is maintained or even increased. Therefore, there is only minimal placental stress and sFLT and PLGF secretion by the placenta are close to normal range. These cases, which represent the overwhelming majority of pre-eclampsia, are now thought to be due to a genetic maternal pre-disposition to cardiovascular disease, which manifests as pre-eclampsia in response to pregnancy-induced stress.

Thus, sFlt-1 and sEng do not serve solely as biomarkers, rather they are responsible for the angiogenic imbalance and generalized endothelial dysfunction characterizing preeclampsia. The new insights into the pathogenesis of this clinical condition will provide great opportunity to improve the care of preeclamptic women before delivery and undoubtedly will lead 1 day to the development of novel strategies for prevention and treatment of the disease. Pipeline clinical trials based on elimination of serum sFlt-1 by means of apheresis yielded promising results indicating that the remedy for this prevalent dangerous entity is within reach (Nakakita et al., 2015; Easterling, 2016).

## Author Contributions

ZA writing the section that deals with the renal aspect of preeclampsia and organizing all sections. JJ writing the introduction and organizing the MS. AJ writing the biochemical biomarkers of preeclampsia. ZAA writing the vascular aspects of preeclampsia and the molecular base underlying the pathogenesis of the diseases, editing the whole MS.

## Conflict of Interest Statement

The authors declare that the research was conducted in the absence of any commercial or financial relationships that could be construed as a potential conflict of interest.
